# Postoperative maternal complications of caesarean section: a cross-sectional study at the Provincial General Hospital of Kananga in the Democratic Republic of Congo

**DOI:** 10.11604/pamj.2024.47.23.40458

**Published:** 2024-01-22

**Authors:** Antoine Tshimbundu Kayembe, Sylvain Mulumba Kapuku

**Affiliations:** 1 Department of Gynecology and Obstetrics, Faculty of Medicine, University Notre-Dame of Kasayi, Central Kasai, Democratic Republic of Congo

**Keywords:** Postoperative complications, caesarean section, General and Provincial Hospital, Kananga

## Abstract

**Introduction:**

the maternal complications of caesarean section make it considered a riskier route of delivery than the vaginal route. The caesarean patient in fact combines the risks of giving birth and those of abdominal surgery. The objective of our study is to determine the epidemiological and therapeutic profile of postoperative maternal complications of caesarean section at the Provincial General Hospital of Kananga from January 1^st^, 2016 to December 31^st^, 2020.

**Methods:**

this is a cross-sectional study of cases of postoperative maternal complications of cesarean section, based on non-probability convenience sampling for case selection. multivariable logistic regression was used in statistical analyses. Our study sample was 302 cases.

**Results:**

the hospital frequency of postoperative complications of cesarean section is 34.12% with the annual average of 60.40 (17.21) cases per year. The postoperative infections are the most frequent complication with more than 52.98% (n=160), treatment is surgical in 59.61% (n=180), the maternal mortality rate due to postoperative complications of cesareans is 5.63% (n=17). Five factors independently associated with postoperative complications of cesarean section were identifying: prolonged labor (aOR: 3.110, 95% CI: 1.040-9.250; p=0.001), defective hygiene of patients (aOR: 1.910, 95% CI: 1.090-10.930; p=0.001), uterine overdistension before caesarean section (aOR: 4.290, 95% CI: 3.320-5.550; p=0.000), multiparity (aOR: 2.070, 95% CI: 1.010-5.210; p=0.006) and emergency cesarean section (aOR: 1.510, 95% CI: 1.250-1.910; p=0.000) in our environment and during the period of our study.

**Conclusion:**

intraoperative complications of ceasarean section constitute a real health problem. These five factors independently associated with postoperative complications of cesarean section could be used for screening of high-risk women in obstetrical consultations during pregnancy monitoring.

## Introduction

Caesarean section is an artificial delivery technique that allows fetal extraction after the surgical opening of the uterus. Its purpose is to save the newborn and the mother in situations of fetal distress or dystocia [1,2].

Its incidence is on the rise worldwide [3,4]. The incidence of caesarean section varies from country to country and from hospital to hospital within the same country [5]. Over the past 30 years, the incidence of caesarean section has increased from 5% to about 25% or even more than 50% in some countries [5]. The risk and safety associated with caesarean delivery differs around the world [6].

Innovations in operating and anesthetic techniques to provide good maternal-fetal safety have made caesarean section a common intervention in obstetrics. But its complications, especially maternal per and postoperative, are not exceptional [2]. Despite these innovations, the rates of maternal complications remain high, sometimes jeopardizing the vital prognosis and the obstetric future of patients. In Africa, some authors have reported the incidence of maternal complications of caesarean section which varies from 10.3% in a Moroccan study to 40.55% in a Guinean study [7-10]. Maternal complications from caesarean section make it considered a riskier route of delivery than the vaginal route. The caesarean patient in fact combines the risks of childbirth and those of abdominal surgery [2,11].

The risk factors for maternal complications of cesarean section described in the literature are: poor patient hygiene, prolonged labor, multiparity, inexperience of the operator or surgeon, obesity or index of body mass greater than 30 Kg/m^2^, low socio-economic level, age of the patient, uterine overdistension, emergency cesarean section, septic conditions of operability [1,8-12].

The lack of epidemiological data on postoperative maternal complications of caesarean section in our community prompted us to conduct this study, the objective of which is to describe the epidemiological, clinical, and therapeutic profile of postoperative complications of caesarean section in the maternity ward of the Provincial General Hospital (PGH) of Kananga from January 1^st^, 2016 to December 31^st^, 2020.

## Methods

**Study design and setting:** this is a cross-sectional study of cases of postoperative maternal complications of caesarean section recorded at the maternity ward of the PGH of Kananga from January 1^st^, 2016 to December 31^st^, 2020. The maternity ward of PGH of Kananga was chosen at because of its situation as the second provincial reference hospital for cases, the presence of trained and experienced staff, and the high attendance of patients who trust its staff.

**Study population:** we used the medical records of patients aged between 16 and 45 years old who underwent caesarean section complicated by postoperative maternal complications and registered at the maternity ward of the Kananga Provincial Hospital from January 1^st^, 2016 to December 31^st^, 2020. Our sampling is non-probability of convenience. The sample size was determined by the limitation of our study in time and space. The following criteria enabled us to include the patients in the study: patients aged between 16 and 45 years old, having undergone a caesarean section complicated by postoperative maternal complications at the maternity ward of the HGP of Kananga from January 1^st^, 2016 to December 31^st^, 2020 and whose medical file was complete. Patients who did not meet these inclusion criteria and had incomplete medical records were excluded.

**Collection of data:** data was collected from operating room registers, maternity registers, maternity patient medical records, and the data collection sheet. The variables of our study are: year of study, age of patients, weight, height, BMI, parity, operator, uterine overdistension, hygiene of patients, type of labor, clinical and therapeutic characteristics of postoperative complications of cesarean section. The data collection was done as follows: we first identified the names of patients who had suffered from postoperative complications of cesarean section in the operating room and maternity registers, then carried out the search for medical records based on the names of identified patients and finally transcribes the data from the medical records of these patients identified in the data collection sheet.

**Definitions:** parity: number of pregnancies having reached 28 weeks of amenorrhea in a woman [13]. Primiparity: presence or history of a pregnancy having reached 28 weeks of amenorrhea in a woman [13]. Pauciparty: the presence of two or three pregnancies having reached 28 weeks of amenorrhea in a woman [13]. Multiparty: story of more than four pregnancies reaching 28 weeks of amenorrhea in one woman [13]. Body mass index (BMI): ratio of weight expressed in kilograms and height in meters squared [13]. Uterine overdistension: uterine height greater than 32 cm for a full-term pregnancy [13].

**Statistical analysis:** data was analyzed using Statistical Package for Social Sciences (SPSS) software version 29. We used the average (SD) to present the quantitative variables and the proportion to present the qualitative variables. The T-student test and the Chi-square test were used to compare averages and proportions between groups respectively. The univariable logistic regression analysis was used to evaluate the strength of the association between observed factors and genital prolapse's appearance. The multivariable logistic regression analysis was used to identify factors associated with genital prolapse among variables with p-value of less than 0.2 in the univariable analysis. A p-value <0.05 was considered statistically significant.

**Ethical considerations:** principles of medical ethics and documentary studies rules have been respected; the data were collected confidentially and treated anonymously. Our study was approved by the local ethics committee of PGHK and registered and its reference number of approval is HGPK/CE/0228/2020.

## Results

**Figure 1:**
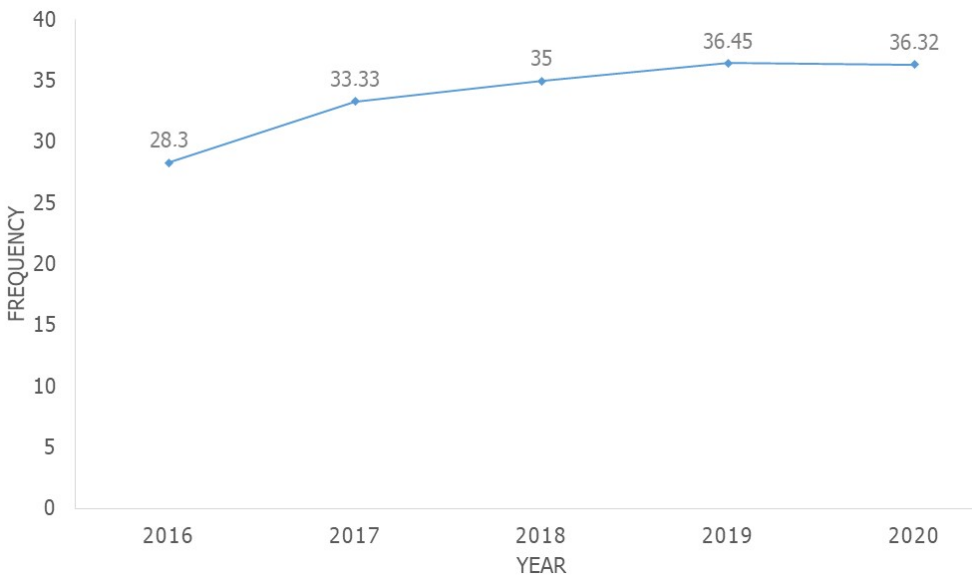
frequency´s evolution of postoperative complications of ceasarean section during our study´s period

**Frequency of postoperative complications of caesarean section during the study period:** we recorded 885 caesareans out of 6060 deliveries at the maternity hospital of the HGP of Kananga during the study period. Of these 885 caesareans, 302 were complicated in the postoperative period, i.e. a hospital frequency of postoperative complications of caesarean section of 34.12%, the evolution of which during the period of our study went from 28.30% in 2016 to 36.32% in 2020 in going through 33.33% in 2017, 35.00% in 2018 and 36.45% in 2019. The average number of cases of postoperative complications of cesarean section was 60.40 (17.21) cases per year during the period of our study and in our environment (Figure 1).

**Clinical and therapeutic characteristics of postoperative complications of cesarean section:** postoperative bleeding was encountered in 136 cases, i.e. 45.03%, parietal and wound infections in 81 cases, i.e. 26.82%, endometritis in 51 cases, i.e. 16.88%, thrombophlebitis in 4 cases, i.e. 1.32%, paralytic ileus in the base of abdominal bloating in 2 cases or 0.66%, pelviperitonitis in 11 cases or 3.64% and generalized peritonitis in 17 cases or 5.63%. As for the established treatment, it was medical in 122 cases, or 40.39%, and surgical in 180 cases, or 59.61%. The post-therapeutic evolution was good or characterized by maternal recovery in 285 cases or 94.37% and by maternal death in 17 cases or 5.63% (Table 1).

**Table 1:** distribution of cases according to clinical and therapeutic characteristics of postoperative complications of cesarean section

Postoperative complications	N=302	%
Hemorrhage	136	45.03
Parietal and wound infections	81	26.82
Endometritis	51	16.88
Thrombophlebitis	4	1.32
Paralytic ileus at the base of abdominal bloating	2	0.66
Pelviperitonitis	11	3.64
Generalized peritonitis	17	5.62
**Treatment initiated**		
Medical	122	40.40
Surgical	180	59.60
**Post-therapeutic evolution**		
Good (characterized by maternal healing)	285	94.37
Maternal death	17	5.63

N: total number of cases; %: percentage

**Factors independently associated with postoperative complications of caesarean:** univariable analyses allowed us to note a significant association between genital prolapse's occurrence and the following factors: age of patients less than 35 years, BMI superior to 30 Kg/m^2^, prolonged labor, defective hygiene of patients, uterine overdistension before caesarean section, multiparity, inexperienced operator and emergency cesarean section (Table 2) (this table presents univariable and multivariable analyses which allowed us to identify factors independently).

**Table 2:** factors independently associated with postoperative complications of cesaeran section

	Univariable analysis		Multivariable analysis	
	aOR (95% CI)	p-value	aOR (95% CI)	p-value
Age of patients less than 35 years (reference: no)	6.220 (3.770-10.260)	<0.001	1.260 (0.040 - 34.980)	0.785
BMI superior to 30 Kg/m^2^ (reference: no)	3.740 (1.870-15.880)	<0.001	3.020 (0.0630 - 14.470)	0.476
Prolonged labor (reference: no)	1.520 (1.234-4.320)	<0.001	3.110 (1.040 - 9.250)	0.001
Defective hygiene of the patient (reference: no)	1.550 (1.360-1.840)	0.005	1.910 (1.090 - 10.930)	0.001
Uterine overdistension before caesarean section (reference: no)	0.020 (0.011-0.041)	<0.001	4.290 (3.320 - 5.550)	<0.001
Multiparity (reference: no)	0.017 (0.002-0.12)	<0.001	2.070 (1.110 - 5.210)	0.006
Inexperienced operator (reference: no)	6.130 (3.810-9.880)	<0.001	0.170 (0.005 - 5.597)	0.240
Emergency cesarean section (reference: no)	0.003 (0.000-0.010)	<0.001	1.510 (1.250 - 1.910)	<0.001

BMI: body mass index

Multivariable analyses identified prolonged labor (aOR: 3.110, 95% CI: 1.040-9.250; p=0.001), defective hygiene of patients (aOR: 1.910, 95% CI: 1.090-10.930; p=0.001), uterine overdistension before caesarean section (aOR: 4.290, 95% CI: 3.320-5.550; p=0.000), multiparity (aOR: 2.070, 95% CI: 1.010-5.210; p=0.006) and emergency cesarean section (aOR: 1.510, 95% CI: 1.250-1.910; p=0.000) as factors independently associated with postoperative complications of cesareans were identifying in our environment (Table 2).

## Discussion

Our study aimed to determine the epidemiological and therapeutic profile of postoperative maternal complications of caesarean section at the Provincial General Hospital of Kananga from January 1^st^, 2016 to December 31^st^, 2020.

The frequency of postoperative maternal complications of caesarean section is 34.12% with the annual average of 60.40 (17.21) cases per year. The postoperative infections are the most frequent complication with more than 52.98% (n=160), treatment is surgical in 59.61% (n=180), the maternal mortality rate due to postoperative complications of cesareans is 5.63% (n=17). Five factors independently associated with postoperative complications of cesareans were identifying: prolonged labor (aOR: 3.110, 95% CI: 1.040-9.250; p=0.001), defective hygiene of patients (aOR: 1.910, 95% CI: 1.090-10.930; p=0.001), uterine overdistension before caesarean section (aOR: 4.290, 95% CI: 3.320-5.550; p=0.000), multiparity (aOR: 2.070, 95% CI: 1.010-5.210; p=0.006) and emergency cesarean section (aOR: 1.510, 95% CI: 1.250-1.910; p=0.000).

The frequency of postoperative maternal complications of caesarean section otherwise called “postoperative maternal morbidity rate of caesarean section” was 34.12% in our environment. Its evolution during the period of our study went from 28.30% in 2016 to 36.32% in 2020 passing through a peak of 36.45% in 2019. Our postoperative maternal morbidity rate of cesarean section is lower than those of Benkirane *et al*. in Morocco [12] and Ugwu *et al*. in Guinea-Conakry [9] which are respectively 63.60% and 40.55%. It is superior to that of Ngowa *et al*. in Cameroon [10] which is 19.70%. Several factors would explain the high rates of postoperative maternal morbidity of caesarean section such as: the absence of modern means of monitoring pregnancy and parturition, the low socio-economic and intellectual level of the populations, the lack of qualified personnel in maternity wards, the under-equipment that characterizes our hospital institutions and the role that ethnoculture still plays in reproductive behavior even in the middle of an urban environment [14-17]. These may also explain ours. The average age of caesareans complicated by postoperative maternal complications of caesarean is 29.32 years (SD 8.99). Our results are lower than those of Benkirane *et al*. in Morocco [12], Trabelsi *et al*. in Tunisia [2] who reported an average age of complicated caesareans of 30.50 years and 30.20 years, and is higher than that of Ngowa *et al*. in Cameroon [10] which is 28.13 years. In the literature, the indications for caesareans are multiple and any obstetric pathology can be one [13,18]. In our case series, we have the following indications: hemorrhagic placenta previa, uterine rupture, fetal-pelvic disproportion, narrowed pelvis, transverse presentation, cord prolapse, hand prolapse, and complicated dynamic dystocia of acute fetal distress.

As for postoperative maternal complications, infections were more encountered in 52.96% of cases in the form of parietal and wound infections, endometritis, pelviperitonitis and generalized peritonitis. Our results corroborate those of Dao *et al*. in Burkina Faso [19], Benkirane *et al*. in Morocco [12], Ngowa *et al*. in Cameroon [10] where postoperative infections predominate. The context in which caesarean section is performed in our climates easily explains this infectious predominance: emergency caesarean section, prolonged premature rupture of membranes with amniotic infection, prolonged labor and defective intraoperative asepsis conditions [10,12,19]. This is also the case in our environment. These infections lead to significant postoperative morbidity which prolongs the duration of hospitalization and significantly increases the financial cost [12]. These results confirm the preponderant role of compliance with intraoperative asepsis and systematic cover antibiotic therapy in the prevention of postoperative infections in hospitals [10,12,19]. This is also valid for our environment.

In these postoperative infections, we noted the predominance of parietal infections (26.82% of cases) followed by endometritis (16.88% of cases). These results corroborate those of de Benkirane *et al*. in Morocco [12], Ngowa *et al*. in Cameroon [10] where parietal infections predominated over endometritis. We noted the thrombophlebitis of lower limbs in 4 cases is a rate of 1.32% in spite of the systematic prevention by the early rise. Our rate is higher than that of Benkirane *et al*. in Morocco [12] which is 0.41%. Several factors including infections, prolonged bed rest, hemostasis disorders, obesity, and multiparity explain the occurrence of thrombophlebitis [12,19]. These may also explain its occurrence in our environment.

Postoperative bleeding was present in 45.03% of cases. This rate is consistent with that of Benkirane *et al*. in Morocco [12] which is 44.70%. The clinical estimate of blood loss being very imprecise, often led to an underestimation of the incidence of bleeding (from 30 to 50%), which would explain the variable rates recorded in the literature. Sarfati proposed a biological assessment based on the drop in hemoglobin and hematocrit [20]. In our study, we considered hemorrhagic caesarean sections where blood loss was judged subjectively important by the operators. These hemorrhages may be due to hemostasis disorders or local placental or uterine factors or secondary to traumatic lesions [10,21-23].

The established treatment was surgical in 59.60% of cases and medical in 40.40% of cases. This can be explained by the high frequency of infections and postoperative bleeding in our environment, the treatment of which is medico-surgical according to the literature [10,12,20-22]. As for the post-therapeutic evolution, maternal mortality linked to postoperative complications of cesarean section was 5.63%, much higher than those of Kinenkinda *et al*. in Lubumbashi (DR Congo) [14], Ugwu *et al*. in Guinea-Conackry [9], by Kemfang *et al*. in Cameroon [10] and Benkirane *et al*. in Morocco [12] which are respectively 1.40%, 3.45%, 1.90% and 4.2%. According to the observations of many authors here in the DR Congo as elsewhere in most developing countries, the high maternal mortality linked to postoperative complications of caesarean section can be due to the low socio-economic and intellectual level of the populations, the lack of personnel qualified in the maternities and the under-equipment that characterizes our hospitals [14-17]. These may also explain ours.

Factors independently associated with postoperative complications of cesareans in our study were: prolonged labor, defective hygiene of patients, uterine overdistension before caesarean section, multiparity, and emergency caesarean section. Our observation corroborates thoses of many authors in the literature [1,8-12,15,16]. Good monitoring and compliance with the partogram during childbirth, and health advice to be given to pregnant women regarding their hygiene during and after childbirth would make it possible to minimize the risk of these complications of cesarean section in our environment as has been advocated Formulu *et al*. in Yaoundé [23].

The weakness of our study is not to have studied the intraoperative complications of cesarean section at the same time while its strength is to be the first to study the epidemiological and therapeutic particularity of postoperative complications of cesarean section and their factors associated in hospital settings of Kananga in Central Kasai in DR Congo.

## Conclusion

It clearly appeared in this study that the frequency of complications of caesarean section was 34.12%, postoperative infections were the most frequent complications in more than half of cases and five factors independently associated with postoperative complications of cesareans were identified: prolonged labor, defective hygiene of patients, uterine overdistension before caesarean section, multiparity, and emergency cesarean section. Our results warrant deepened studies upon caesarean section in order to allow scientists to raise awareness upon caesarean section´s studies and to improve its pratices in our milieu. These data could be used for screening of high-risk women in obstetrical consultations during pregnancy monitoring.

### What is known about this topic

- *The purpose of the cesarean section is to save the newborn and the mother in situations that endanger the fetal and maternal vital prognosis;*
- *Postoperative maternal complications of caesarean section make it considered as a more risky way of childbirth than the vaginal way;*
- *The lack of data on the frequency, types, treatment, and factors associated with postoperative complications of caesarean-section in hospitals of Kananga, in the DR Congo*.

### What this study adds

- *The frequency of postoperative complications of cesarean section is of 34.12% and post-operative infections are the most frequent type of postoperative complication of cesarean-section;*
- *Their five factors independently associated were identifying: prolonged labor, defective hygiene of patients, uterine overdistension before caesarean section, multiparity, and emergency cesarean section;*
- *These data could be used for screening of high-risk women in obstetrical consultations during pregnancy monitoring*.
